# Predicting suicidal thoughts and behavior among adolescents using the risk and protective factor framework: A large-scale machine learning approach

**DOI:** 10.1371/journal.pone.0258535

**Published:** 2021-11-03

**Authors:** Orion Weller, Luke Sagers, Carl Hanson, Michael Barnes, Quinn Snell, E. Shannon Tass

**Affiliations:** 1 Department of Computer Science, Johns Hopkins University, Baltimore, Maryland, United States of America; 2 Department of Computer Science, Brigham Young University, Provo, Utah, United States of America; 3 Department of Biomedical Informatics, Harvard University, Cambridge, Massachusetts, United States of America; 4 Department of Statistics, Brigham Young University, Provo, Utah, United States of America; 5 Department of Public Health, Brigham Young University, Provo, Utah, United States of America; Columbia University, UNITED STATES

## Abstract

**Introduction:**

Addressing the problem of suicidal thoughts and behavior (STB) in adolescents requires understanding the associated risk factors. While previous research has identified individual risk and protective factors associated with many adolescent social morbidities, modern machine learning approaches can help identify risk and protective factors that interact (group) to provide predictive power for STB. This study aims to develop a prediction algorithm for STB among adolescents using the risk and protective factor framework and social determinants of health.

**Methods:**

The sample population consisted of more than 179,000 high school students living in Utah and participating in the Communities That Care (CTC) Youth Survey from 2011-2017. The dataset includes responses to 300+ questions from the CTC and 8000+ demographic factors from the American Census Survey for a total of 1.2 billion values. Machine learning techniques were employed to extract the survey questions that were best able to predict answers indicative of STB, using recent work in interpretable machine learning.

**Results:**

Analysis showed strong predictive power, with the ability to predict individuals with STB with 91% accuracy. After extracting the top ten questions that most affected model predictions, questions fell into four main categories: familial life, drug consumption, demographics, and peer acceptance at school.

**Conclusions:**

Modern machine learning approaches provide new methods for understanding the interaction between root causes and outcomes, such as STB. The model developed in this study showed significant improvement in predictive accuracy compared to previous research. Results indicate that certain risk and protective factors, such as adolescents being threatened or harassed through digital media or bullied at school, and exposure or involvement in serious arguments and yelling at home are the leading predictors of STB and can help narrow and reaffirm priority prevention programming and areas of focused policymaking.

## Introduction

Suicide is the 10th leading cause of death among adults [1] and the 2nd leading cause of death among adolescents [2] in the United States (US). Suicidal thoughts and behavior (STB) are significant public health challenges contributing to suicide. As such, a greater understanding of the most important risk and protective factors related to STB is critical for improving prevention and treatment efforts. However, in recent years, less attention has been given to suicidal thoughts as practitioners have been focused primarily on suicides and attempts [3]. In addition, more research is needed to understand the specific risk factors associated with STB [4].

Among adolescents, single research studies have been conducted to understand what contributes to and reduces the risk of suicide. These studies have identified many risk factors including, but have not been limited to, drug use [5] emotional self-efficacy [6], and crisis in meaning as risk factors contributing to STB [7]. Factors associated with resiliency have also been shown to be protective against adolescent suicidal behavior [8]. As a result of these types of studies, a number of organizations have compiled lists of suicide risk factors and warning signs meant to assist practitioners in their prevention and treatment work [4]. Despite this body of work over the past 50 years, STB researchers have argued that meta-analysis findings show that previous studies fail to generalize (predictive rates barely above chance), their methodologies are limited, and they contain small scopes of analysis [4].

While the field of prevention science has historically focused on the identification of specific risk factors associated with an outcome (e.g., suicide), less has been done to explore which combination of risk as well as protective factors are associated with an outcome such as suicide in large samples [9]. Machine learning approaches have been recognized as ideal for such a task [10] and shift the focus away from limited linear bivariate risk prediction models to multivariate risk algorithms that identify patterns in large amounts of data that learn an outcome of interest [4]. These techniques are advantageous for enhanced predictive accuracy and have the potential to create nonparametric predictive risk profiles for adolescent STB.

### Theoretical framework

To date, addressing adolescent social morbidities such as substance abuse, mental health, and violence has largely utilized the risk and protective factor framework [11]. This framework provides the theoretical scaffolding around which the Communities That Care (CTC) prevention programming [12] is based on and suggests that communities must give careful attention to reduce adolescent social morbidities to a specific group of protective and risk factors. The CTC program involves assembling community stakeholders to set priorities and leverage a variety of combined resources to address community needs. Protective factors that are important for adolescent social development include opportunities for prosocial involvement, rewards for prosocial involvement (recognition), skills, strong social bonds and attachment, healthy beliefs, and clear standards [13]. Risk factors have been identified across four ecological domain areas (peer/individual, school, family, and community) that disrupt the social development of adolescents [11, 14, 15].

A comprehensive synthesis of CTC randomized controlled trials (RCTs) and controlled prospective studies (CPSs) [16–26] demonstrated reduced incidence and prevalence of adolescent delinquency and substance use as a result of communities working to address adolescent risk and protective factors. Generally, these studies reported significantly higher levels of protection in the community, school, and peer/individual domains, but not in the family domain. Specific examples within those domains include prosocial involvement, social skills, neighborhood involvement, commitment to school, and healthy beliefs and clear standards. Another study in 2015 found similar results and identified several protective family factors such as feeling close to one’s mother and recognizing prosocial involvement in their CTC protection factor intervention [20].

Social factors that influence adolescents’ health have also been referred to as the social determinants of health (SDH). The SDH are defined as “the conditions in which people are born, grow, live, work and age” [27]. These conditions are shaped by the families and communities in which adolescents are exposed as well as the distribution of economic and other resources available based on policy choices. Research indicates that SDH structural factors such as national wealth, income inequality, and access to education are the most important determinants of adolescent health [28].

Over the past decade, machine learning research has shown that non-parametric methods (such as neural networks, random forests, and other methods) are often better suited to handle large and complex data, where the associations between factors may not be linear. Although these methods provide strong results, they are often harder to explain, leading to a lack of use in fields where explainability is paramount. However, the last few years have shown great progress in machine learning explainability, creating techniques for providing explanations for these algorithms. While others have developed prediction models for adolescent suicide in Korea using general health behavior data [29], no identified study has used machine learning to develop a risk profile of adolescent STB using the risk and protective factor framework [11] and SDH. As such, the purpose of this study was to leverage machine learning techniques to determine which combination of risk factors, protective factors, and SDH factors are most highly associated with STB among adolescents. Such an SDH adolescent risk profile would help more efficiently guide prevention efforts.

## Methods

### Data collection and preparation

The state of Utah routinely administers a questionnaire to adolescents in the 6th, 8th, 10th, and 12th grades to monitor the adolescent population. This survey, the Student Health and Risk Prevention (SHARP) Statewide Survey (Utah Department of Human Services, 2021), is given every 2 years in most public and certain charter school districts across Utah. This survey is also called the Communities That Care (CTC) Risk and Protective Factor Youth Survey or the Prevention Needs Assessment (PNA) Survey, depending on where it is administered [30]. This instrument’s primary goal is to measure the need for prevention services related to adolescent social morbidities such as substance abuse, antisocial behavior, and violence.

The PNA survey contains questions including a comprehensive range of topics, including basic demographics, family life, past behavior, community involvement and perception of norms, detailed information on school involvement and behavior, drug usage, gambling, religion, and antisocial behavior. Demographic information (grade level, gender, ethnicity, etc.) for each year’s survey can be found in Table 1. The honesty of respondents are assessed through three questions, designed by the SHARP creators: how old respondents were when they used a fake non-existent drug, how many times they used the fake drug in the last 30 days, and whether they said they were honest while filling out the survey. Surveys from respondents that indicated they used the fake drug (meaning they had lied or were untrustworthy) or who indicated they were not honest were removed from the analysis. The survey questions are randomized, making the question order different between years and even between the two versions that are administered yearly. Although this survey randomization is a great feature for reliability, it makes the data preparation process difficult.

**Table 1:** Demographic information for each survey year.

Variable	2011	2013	2015	2017	Total
Female	13754 (51.6)	26068 (51.3)	24346 (51.2)	25661 (51.5)	89829 (51.4)
Grade 6	7777 (29.2)	13923 (27.4)	13274 (27.9)	15869 (31.9)	50843 (29.1)
Grade 8	6709 (25.2)	14040 (27.6)	12932 (27.2)	14922 (30.0)	48603 (27.8)
Grade 10	5659 (21.2)	10816 (21.3)	10064 (21.2)	10737 (21.6)	37276 (21.3)
Grade 12	4949 (18.6)	8358 (16.4)	7389 (15.5)	8292 (16.6)	28988 (16.6)
Hispanic	3852 (14.5)	8508 (16.7)	7747 (16.3)	8857 (17.8)	28964 (16.6)
Non-Hispanic Black	583 (2.2)	1234 (2.4)	1085 (2.3)	1212 (2.4)	4114 (2.4)
Non-Hispanic White	20552 (77.1)	38097 (74.9)	36187 (76.1)	37180 (74.6)	132016 (75.5)
Other Race and Ethnicity	1817 (6.8)	3373 (6.6)	2863 (6.0)	2903 (5.8)	10956 (6.3)
Urban	38260 (72.7)	NA	NA	19366 (76.8)	NA
Non-Religious	3678 (13.8)	6999 (13.8)	7187 (15.1)	9736 (19.5)	27600 (15.8)
Father Living in the Home	20059 (75.3)	38359 (75.4)	36549 (76.9)	38464 (77.2)	133431 (76.3)
Total Respondents	26651 (15.2)	50844 (29.1)	47550 (27.2)	49820 (28.5)	174,865 (100)

Demographic results are shown in “n (% of population)” form. NA indicates results that are not available for the given year. Demographics align roughly with general Utah demographics.

To organize the data, we collected the question lists for all years of PNA surveys from 2011 to 2017. The question lists were compared, matched, and carefully validated by both humans and computers to make sure the questions aligned from year by year. Doing so allowed for the development of a consolidated “concordance,” mapping questions to question numbers throughout different years and versions. This provided us with easy access to extract the question responses from each year-and-form dataset to create one combined dataset from all years.

Data associated with SDH were also gathered from the American Community Survey (ACS), an ongoing yearly survey containing detailed demographic information by ZCTAs (roughly corresponding to zipcodes). This information included marriage/divorce rates, racial makeup, labor force details, average household information, income percentages, and educational background percentages, along with many more. Each year of ACS data was processed together into a single dataset: where each row contained the demographic information for a particular year and ZCTA. The ACS dataset was then added to the PNA combined dataset, joining on zipcode to provide further demographic information for the analysis.

In order to understand STB, the following three questions were combined into one binary composite variable of STB with outcomes “has STB” or “does not have STB.” This was done by classifying each response as “has STB” if they answered yes (or ‘1+ times’ in the case of a number of incidents question) to any of the following questions: “During the past 12 months, did you ever seriously consider attempting suicide? (Yes/No),” “During the past 12 months, did you make a plan about how you would attempt suicide? (Yes/No),” and “During the past 12 months, how many times did you actually attempt suicide? (0,1,…,6+ times).”

**Fig 1:**
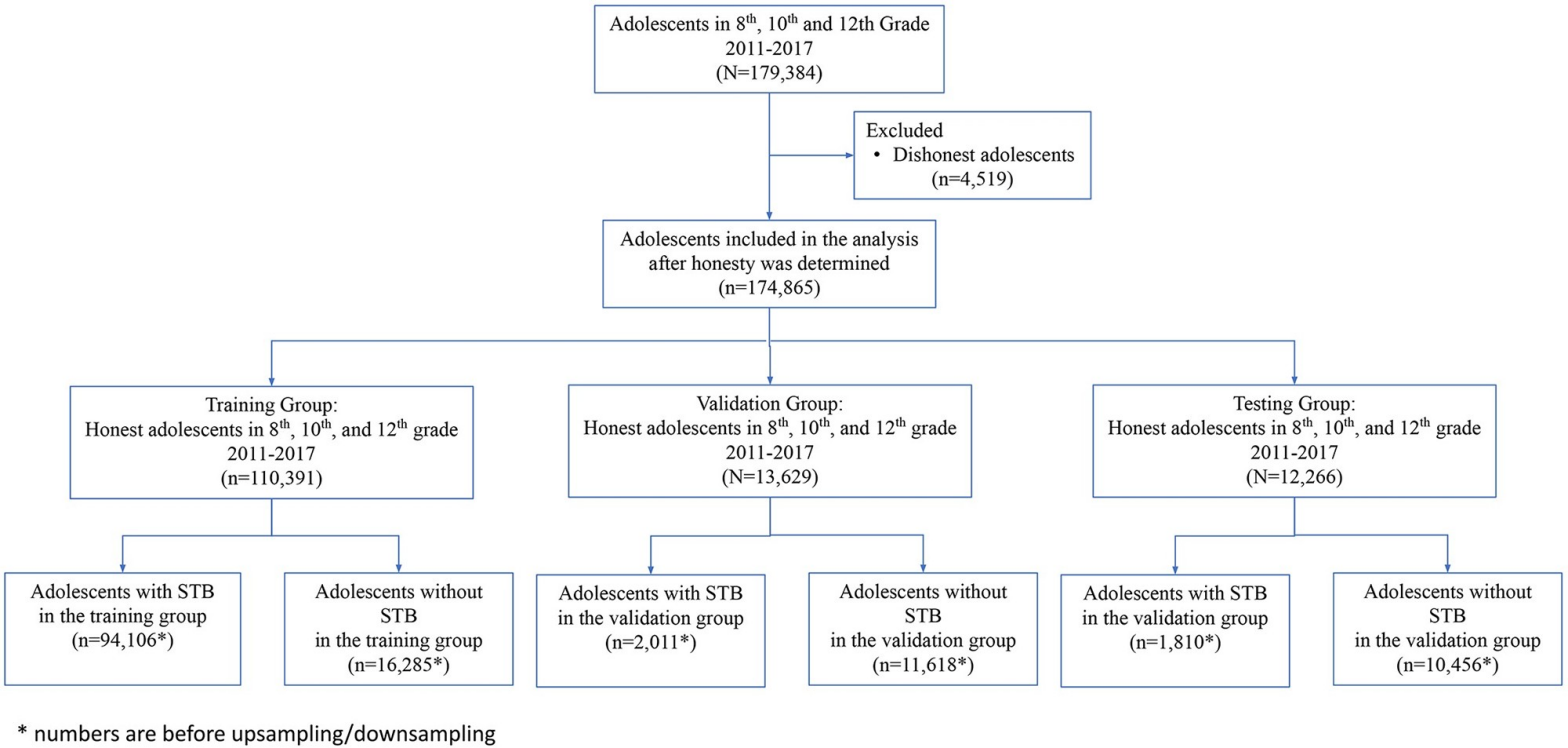
Flow chart of the predictive modeling process. Starting with the initial dataset, we show the process of how we trained and validated our models.

The final dataset contained 179,384 adolescent respondents in 6th, 8th, 10th, and 12th grades with 7900 columns of demographic and survey data. A total of 4,519 adolescents were excluded due to dishonesty leaving 174,864 respondents included in the analysis. A total of 51.4% were female, 29.1% in 6th grade, 27.8% in 8th grade, 21.3% in 10th grade, 16.6% in 12th grade, 75.5% white, 2.4% black, 16.6% Hispanic, and 6.3% other race/ethnicity (see Table 1). The dataset was divided into a roughly 80-10-10 percent split, with a training group (110,391 respondents), a validation group (13,629 respondents), and a testing group (12,266 respondents). This process is shown in Fig 1. To better facilitate training on the imbalanced dataset, we upsampled the minority class in the training and validation sets. We further downsampled the majority class in the test split for clarity in the accuracy metric.

### Models

Modeling was conducted with a variety of machine learning algorithms using the scikit-learn library [31]: K-Nearest Neighbors, Naive Bayes, Logistic Regression, and DecisonTreeClassifier, as well as XGBoost [32] and LightGBM [33]. Models were trained and tuned on the train and validation splits, evaluating only once on the held-out test set for our final numbers. Feature importance (prediction) was determined using the recently introduced SHapley Additive exPlanations (SHAP) metric [34] for tree models. All analysis was completed using Python.

Using the best performing LightGBM model, the most important features (i.e., questions) were analyzed using SHAP tree-based analysis [34]. This method was designed to test the predictions of tree-based models: at a high level this means asking the same question for every prediction and feature, “How does prediction i change when feature j is removed from the model?” This method shows us how influential each survey question is to our models predictions. Since LightGBM results can depend on the random seed used for initialization, we created 100 different lightGBM models, running SHAP analysis on each one. We found the most important questions to be consistent across random seeds, with the top 9 questions appearing in the top 10 in all 100 models and the 10th question appearing in the top 10 90% of the time (and appearing in the top 11 questions 100% of the time). All SHAP figures shown are the average of the 100 seeds, for consistency.

We also perform more fine-grained analysis of several factors, in order to isolate which questions the model is using for a given sub-population. We examine self-reported gender (male, female) and age (10-14 year olds vs 15-19 year olds, roughly middle vs high school), subsetting the full data by the target population and re-training/analyzing the model for that demographic. We also perform a targeted analysis of the outcome variable, separating the STB variable into its three sub-questions (see Section “Data Collection and Preparation”) and re-running the analysis with each binarized sub-question as the target variable instead of the combined STB variable.

## Results

**Fig 2:**
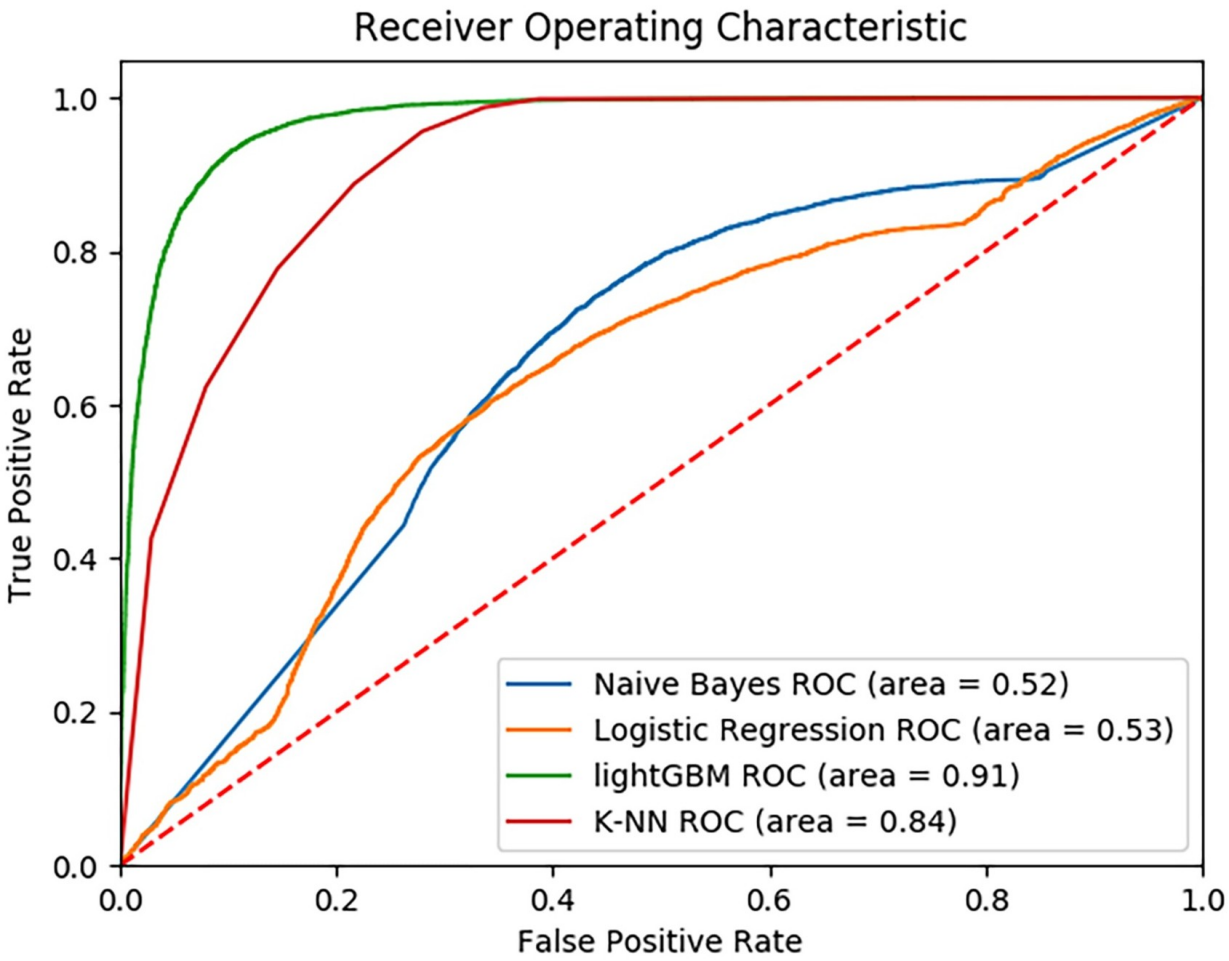
Receiver operating characteristic curve for suicidal thoughts and behavior prediction. We see that the LightGBM model was the highest performing model. Other tree-based models are omitted for clarity, as they performed worse than LightGBM. Higher scores (with curves closer to the upper left corner) are better.

The tree-based model (LightGBM) delivered strong predictive results and out-performed other methods when predicting whether a student exhibited STB (accuracy of 91% on the held-out test set). Due to its strong predictive power, this LightGBM model was used to investigate feature importance and determine which variables (questions from the CTC Survey) had the greatest impact on the model predictions. For more detailed results, Fig 2 illustrates the Receiver Operating Characteristic plots of various machine learning methods tested. Other less sophisticated models, such as Logistic Regression and Naive Bayes underperformed, with scores of 0.53 and 0.52, respectively.

**Fig 3:**
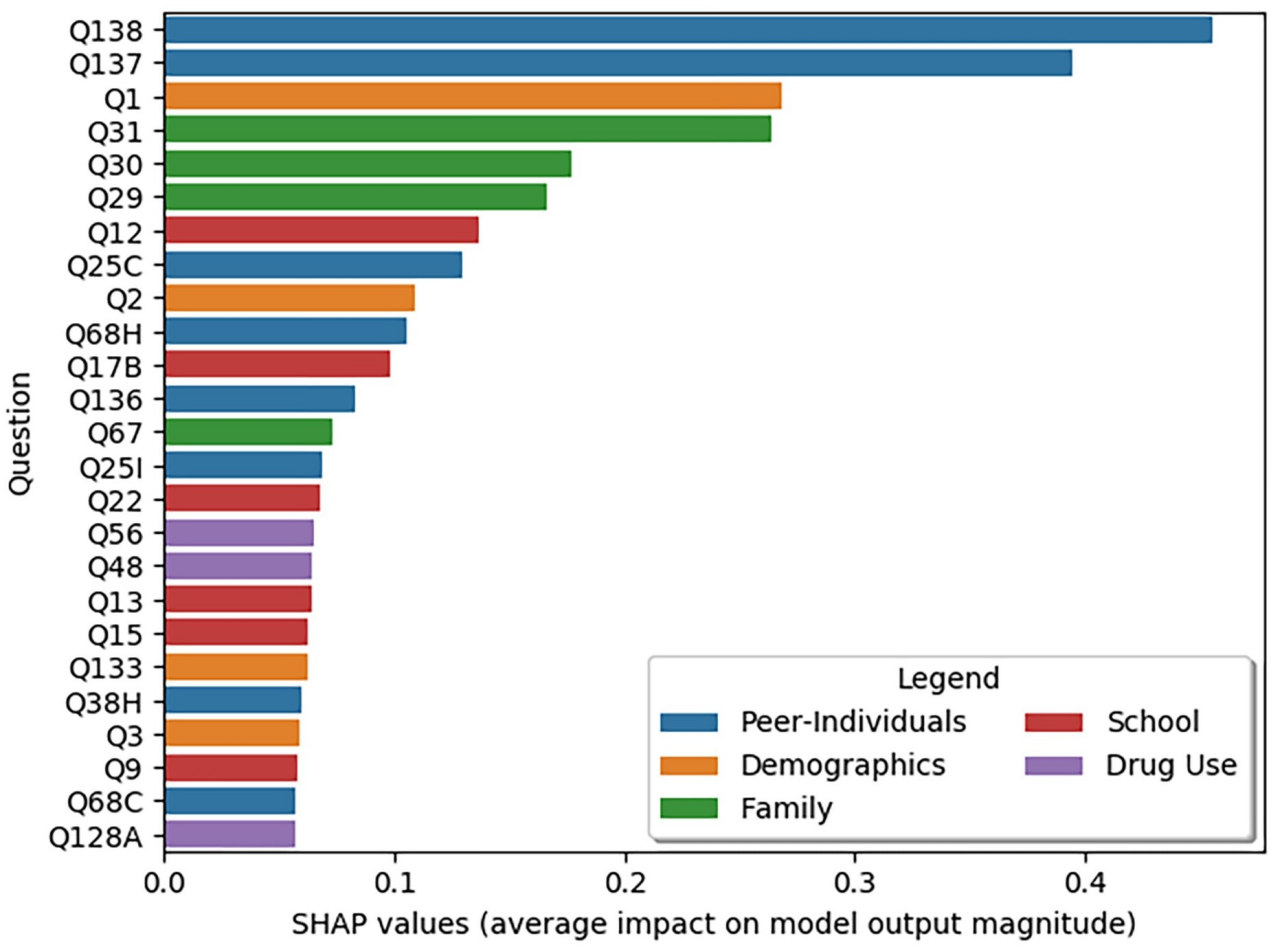
SHAP plots showing feature importance for predicting STB. Note that scores are relative to this particular dataset, with larger scores indicating higher influence.

**Fig 4:**
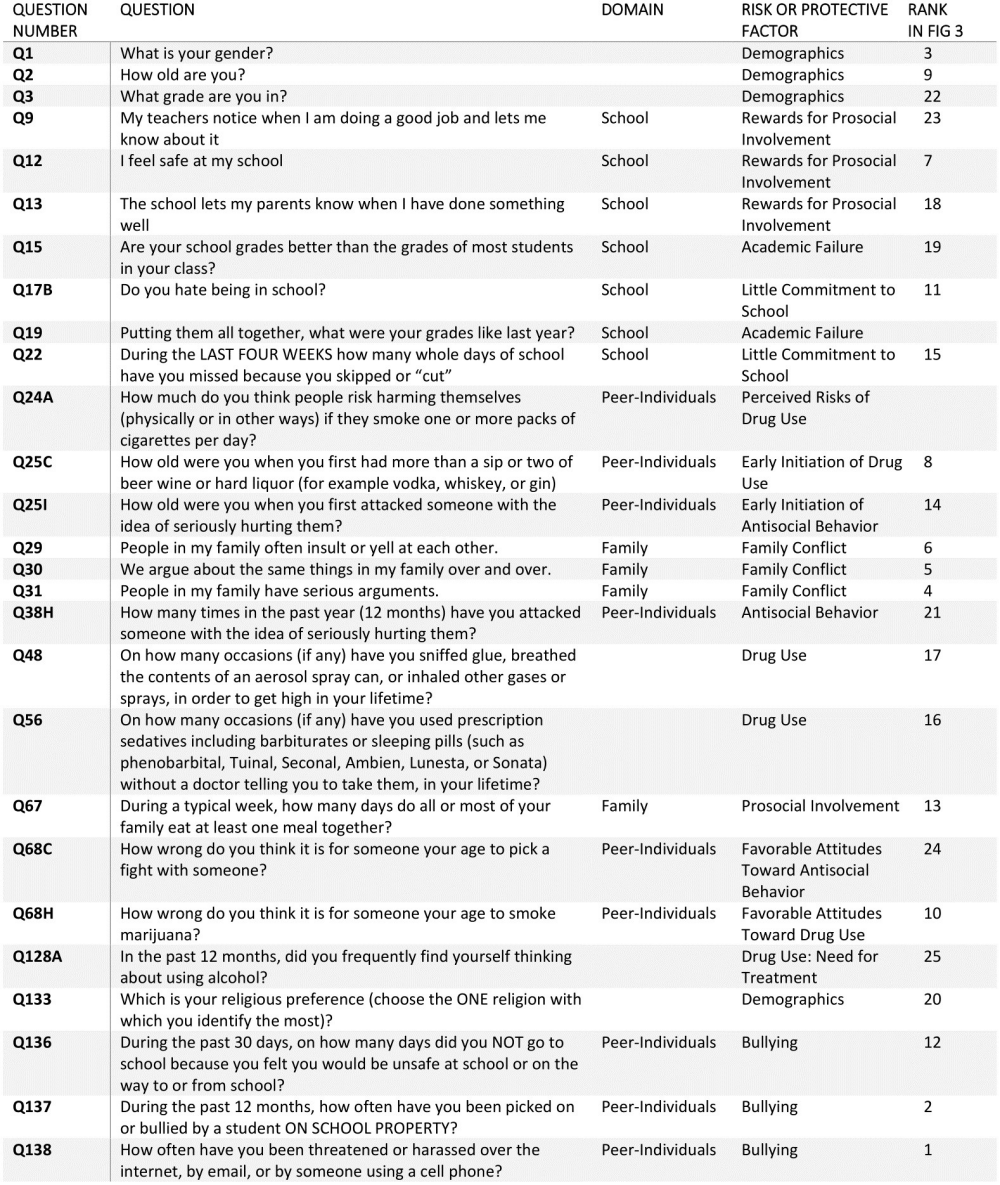
Most predictive questions for adolescent suicidal thoughts and behavior (non-ranked). A table of the most predictive questions, along with which domain and risk and predictive factor they involve, if applicable.

In Fig 3, SHAP importance values are shown for the most important 25 questions (where greater values correlate with greater influence in model predictions). SHAP values indicate the relative importance of features in the predictive model, meaning that there is no standard cut-off. However, after the top ten questions we see that the value increase for including each additional question shows diminishing returns, so we restrict our in-text analysis to the top ten features. These top ten predictive factors of STB include: being threatened or harassed through digital media (Q138), being picked on or bullied at school in the past 12 months (Q137), gender (Q1), being in a family where there are serious arguments (Q31), being in a family that argues about the same things over and over (Q30), being in a family that yells and insults each other (Q29), feeling safe at school (Q12), how old they were when they first had more than a sip of alcohol (Q25C), their age (Q2), and whether they think it’s wrong for someone their age to smoke marijuana (Q68H, note that marijuana is illegal in the state of Utah) (see Fig 3). Of these top ten most predictive factors for STB, four were in the peer-individual domain, three were in the family domain, two were demographic, and one was in the school domain (Fig 3). Fig 3 shows the scores of the SHAP values of the most important features (i.e. questions) and full text for these questions can be found in Fig 4. No SDH factor from the census data contributed significantly to the model’s prediction and listed among the top 25 most predictive factors.

Additional results relative to demographic characteristics revealed that females were more likely to experience STB (17.7% female, 10.8% males). Similarly, when the dynamics between rural and urban areas were examined, 14.8% of those in urban areas had STB compared to 13.2% for those in rural areas. Additionally, those who did not have a father in the home were 72.6% more likely to have STB than those that did (21.2% vs 12.3%). Those in 10th grade or above are 43.8% more likely to have STB (17.5% of the population vs 12.2%). A total of 84.2% of respondents were religious and 15.8% non-religious with a total of 24.7% of non-religious respondents experiencing STB compared to 13.7% of religious respondents. Gender and age however emerged as the only demographic variables in the top ten most predictive factors.

**Fig 5:**
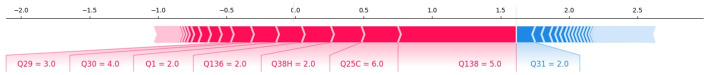
A SHAP force plot of a single individual. This method examines the factors that influenced the model for prediction on a single individual, showing questions that led the model to think they are more likely to have STB in red and questions that led the model to think they are less likely to have STB in blue. A decision boundary of more than zero indicates that the model predicts that they have STB. In this example, you can see that their answer to Q138 of 5 (frequent internet harassment), their answer to Q25C of 6 (early alcohol usage), and their answer to Q38H of 2 (violent activity) led the model to predict that they have STB. This allows for easy interpretability of the model’s results, making it more trustworthy and transparent.

**Fig 6:**
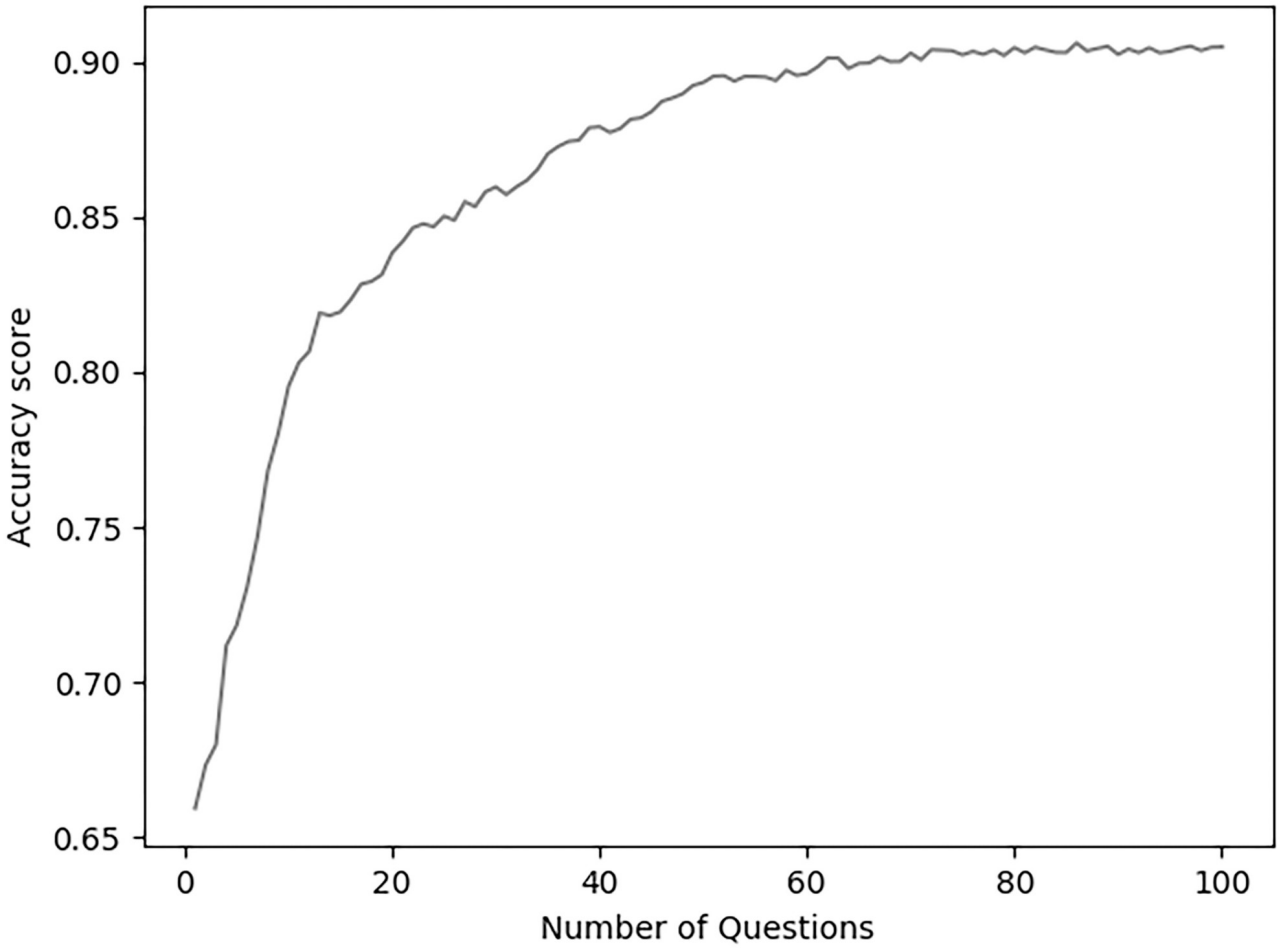
Accuracy scores using the Top-N questions in a cumulative fashion. Results for average model performance of the LightGBM model using the N top questions, along the X-axis. As the number of questions given to the model increases, so does the accuracy.

The SHAP method of analysis also allowed for the interpretability of a single instance, further verifying the results. A prediction of a respondent labeled “has STB” was randomly selected. Fig 5 illustrates which questions influenced the model to classify the respondent as “has STB” including early adolescent use of alcohol, violent behavior, and online harassment. Questions in the survey were also examined as to how they contributed to the level of accuracy (see Fig 6). The analysis revealed that with 20 questions, the model achieved 84% accuracy for STB prediction (which is approximately 92% of the performance that the model gets with the full dataset), while with only 10 questions it scored 79% (80% of the full dataset performance).

### Fine-grained analysis

As discussed at the end of the Methods section, we also examine different aspects of the population in order to isolate what the most important questions are for different demographics. In this section, we discuss how the SHAP analysis for these sub-populations compare to the full analysis. Similar to the full analysis, we discuss the top 10 most important questions, as that is when including additional questions shows diminishing returns in terms of the SHAP importance score. We note that overall most of the top questions remain the same, although some change their order relative to each other: 9 of the top 10 relevant questions for the full analysis are present in each sub-analysis except for the “Suicide Attempt” target variable, which only has 7 of the 10 top questions.

#### Gender

**Fig 7:**
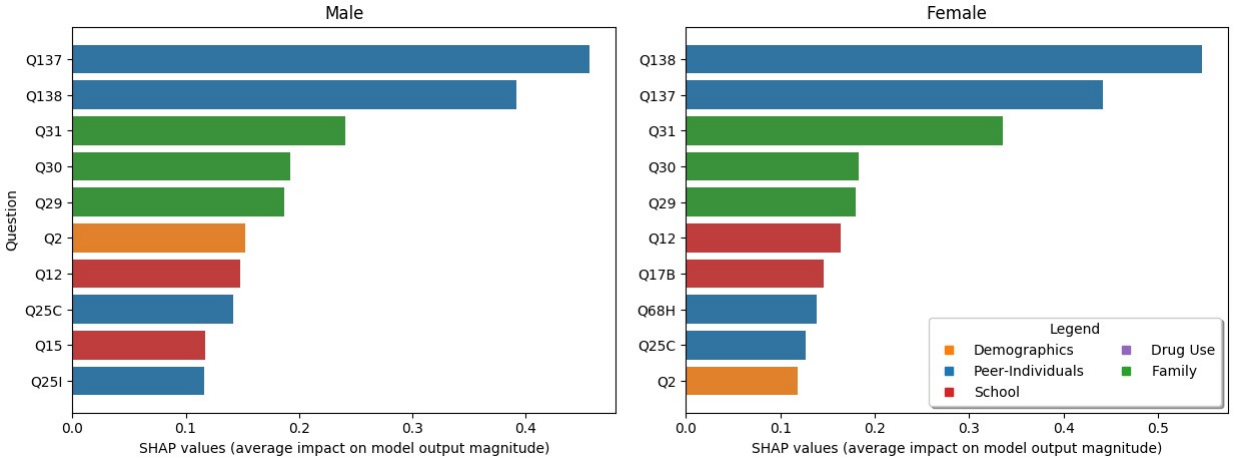
The top 10 most important questions for males vs females. Note that compared to Fig 3 the majority of the questions are the same, however, there are a few slight differences described in the the main text, such as age (Q2), physical aggression (Q25I) and hating school (Q17B).

Examining the male-only model (Fig 7), we find that the major differences are with age, physical aggression, and in-person harassment (rather than online). The age question (Q2) has shifted from the #9 most important question to #6. Furthermore, a question about being involved in physical aggression (Q25I) rises to the top 10 and physical bullying (Q137) changes places with online bullying (Q138) to become the #1 most important question. For females (also in Fig 7), online bullying remains the #1 predictor, while whether they hate school (Q17B) rises into the top 10 and their views on antisocial behavior (underage marijuana usage) rises from #10 to #8. We note that for both models, question #1 (Gender) drops out of the top 10 since all respondents genders are the same in the sub-analysis by design.

#### Age

**Fig 8:**
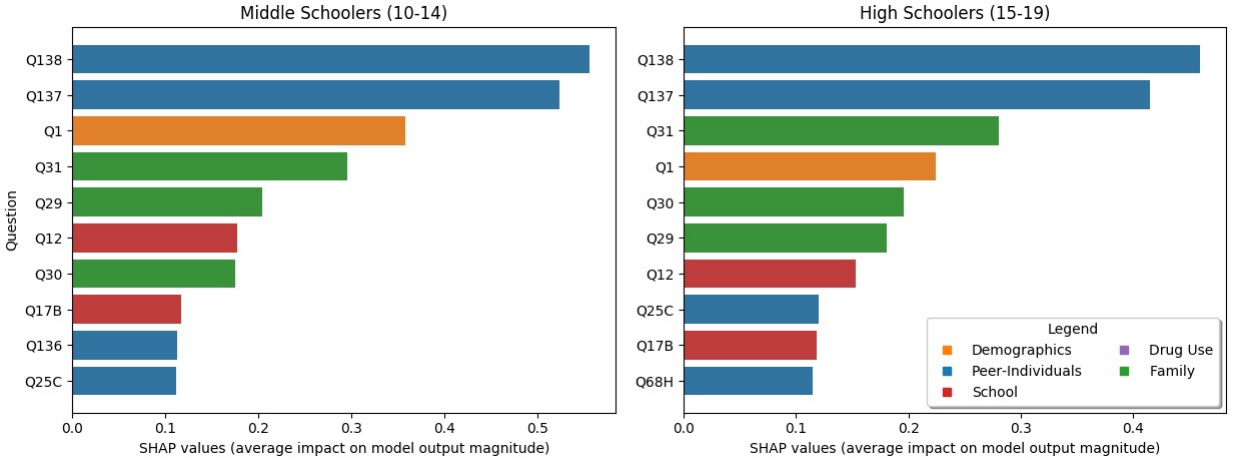
The top 10 most important questions for middle and high school respondents. Note that compared to Fig 3 the majority are the same, however, there are a few slight differences such as the addition of Q17B to both and the addition of Q126 to middle schoolers.

When we split the population into two groups (Fig 8), roughly middle school (10-14) and high school (15-19) we find mostly similar results to the main analysis. Minor differences include the addition of Q17B into both (whether they hate school), the addition of whether they have skipped school because they felt unsafe (middle schoolers, Q136), and for high schoolers whether their family has serious arguments (Q31) rises from #4 to #3. Note that age (Q2) does not show up in the top 10, as the age variable was already used to separate the groups. Altogether, we find that the main conclusions remain the same as the full analysis.

#### Level of suicidal thoughts and behavior

**Fig 9:**
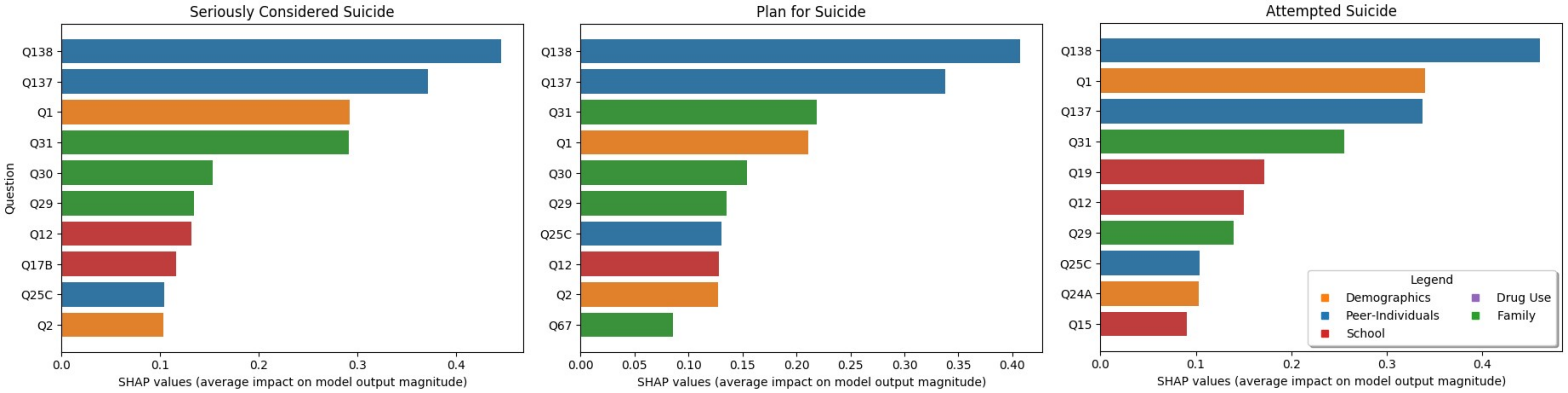
The top 10 most important questions for the varying levels of suicidal thoughts and behavior. Note that compared to Fig 3 the majority are the same. For the target question about “Attempted Suicide” we see the largest difference, with the focus shifting more to the importance of gender (Q1) and school performance (Q15 and Q19).

If we examine the different levels of STB (Fig 9) we see that as the risk increases the questions about gender (Q1), grades (Q15 and Q19) and their views on alcohol/drug use (Q24a and Q25C) become more important predictors. This is especially true in the “Attempted Suicide” target variable where Q1 (Age) becomes the second highest predictor and Q19 (“what were your grades like last year”) rises from outside the top 10 to become the #5 most important question. The difference from the full analysis is largest for the “Attempted Suicide” target where only 7 of the top 10 questions are in the top 10, whereas the other two targets have 9/10 of the top questions from the full analysis.

## Discussion

This studies purpose was to apply machine learning methods to determine which risk factors, protective factors, and SDH factors from the corpus of all PNA and ACS questions are most predictive of adolescent STB. The current study’s value identifies a non-parametric risk profile of the most important factors associated with adolescent STB, using advanced machine learning methods that can handle large data and complex interactions in the global context of all variables. These findings reflect the ranked predictions of STB. To date, no identified study has used machine learning to identify risk profiles for adolescent STB using data associated with the risk and protective factor framework and SDH.

Compared to other models, LightGBM held the highest performing predictive power. Similar research work in this area reported that their top-performing models had accuracy scores in the high 70s for their adolescent risk behavior data [29], but their work did not employ LightGBM. Unlike many other non-parametric machine learning models such as neural networks, this model provides interpretable classification with high accuracy, essential for machine learning techniques that affect human decision-making for STB risk.

Findings revealed that the top ten factors that were most predictive of adolescent STB, rank-ordered by predictability, included: (1) being threatened or harassed over the internet, (2) picked on or bullied by a student at school, (3) gender, (4) involved in serious family arguments, (5) involved in family arguments about the same things over time, (6) belonging to a family that often insults or yells at each other, (7) feeling safe at school, (8) age when more than a sip of alcohol was had, (9) age, and (10) attitude regarding marijuana use.

The top two most predictive risk factors for STB were in the peer-individual domain and included whether adolescents were threatened or harassed through digital media and whether they were being picked on or bullied at school. Previous research has demonstrated the link between cyberbullying and mental health problems using the PNA framework [35]. Additionally, six different meta-analyses have demonstrated that any involvement in bullying (bullying victimization, bullying perpetration, and bully/victim status) is associated with adolescent STB [36]. Findings from the current study demonstrate that internet harassment and school bullying were most predictive (particularly online bullying for females and in-person bullying for males) in relation to the numerous other factors associated with this adolescent STB risk profile.

Specific predictive demographic questions for STB risk that emerged in the top ten included gender and age. Females and older students were found to be at the highest risk for STB compared to males and younger students. Previous research on gender and adolescent suicide has found that the rates of suicide are higher among males (corroborated by the predictive power of gender on attempted suicide in Fig 9), while suicidal behavior is higher among females [37]. Additionally, suicidal thoughts tend to be higher among female adolescents [38]. Our study identified that older age among school-attending adolescents is also among the top predictors of STB risk. This risk rises in the 9th or 10th grade, likely around 15 or 16 years of age. This finding is similar to other studies [39, 40]. These findings point to the importance of continuous developmentally sequenced suicide prevention that is also gender-specific [41].

Our study also showed that student’s perspectives of their grades provides strong predictive power in predicting suicide attempts. Research from the 2015 Youth Risk Behavior Study has shown that students who feel successful at school are less likely to consider, plan, or attempt suicide [42]. School health professionals, state officials, and other stakeholders should target suicide prevention activities or provide grade remediation to assist students at risk.

Three family domain risk and protective factors emerged within the top six most predictive. It is well documented that adolescent development is enhanced through families that provide support, positive communication, family boundaries, cohesion, parents who are involved in school, and establish high expectations [43]. Family communication, especially among male adolescents, has previously been identified as a significant predictor of suicidal thoughts [44]. Findings from the present study lend additional support to the importance of the family context in adolescent development.

Social determinants of health did not appear as a top-ranked predictor of STB. This may be because SDH are typically more distal influencers of health outcomes than the predictors outlined in the risk and protective factor framework. Our predictive model identifies more proximate or immediate predictors for individuals who are prone to STBs. Overall domains such as negative school settings, peer-influence, and family connections appear more problematic than SDH. This finding does not discount the value of SDH because, for example, negative school settings and poor peer and family relations are often more deeply rooted in SDH. So, while prevention programming may focus on the key ranked areas of this study, the policy implications of SDH continue to have significance for stemming the tide for STB.

The implications of this research are important for prevention programming and policies related to adolescent STB. Prevention program specialists and other policymakers can use the STB risk profile and its associated rankings to prepare services, resources, and assessments aimed at school, community, and family settings. As such, these findings also have important implications for prevention policy and resource allocation. For example, continuing and growing a focus on bullying on social media and bullying or intimidation at school or other community settings are prime areas for policy response. Also, given that three family domain risk factors emerged in the top six, family-focused interventions should be a priority of STB prevention programming, especially those that focus on strengthening family functioning. Particular attention should be given to better recognize families as a critical setting of public health practice [45], including the promotion of evidence-based parenting interventions in primary care settings [46].

While not directly associated with the primary aim of the present study, a portion of the highest predictive questions in the model was tested to determine if they could be used in a 10 or 20-item questionnaire. Reasonable comparability to predict STB with a short survey compared to the performance that the model gets with the full dataset was confirmed (84% accuracy with only 20 questions vs 91% with all questions). These results support the notion that a shorter survey could be created and more reasonably distributed without having students take a multi-hundred question survey. Such a survey could be used to inform STB prevention programming more expeditiously. For example, these techniques could enable prevention researchers and evaluators to create questionnaires that could assess risk and provide explanations for their predictions using advanced and transparent machine learning techniques.

### Limitations and future research

Due to the limited accessibility of data sharing, our results are limited to Utah specific findings, as we were not able to compare with other states. However, the methods and processes applied in this paper are applicable to data from all states. We hope that this research will open up the potential for improved cross-state comparisons through data sharing. We note that privacy concerns are a major barrier in these types of analysis, to prevent researchers from comparing individual schools, but geographical comparisons by zip code (such as our analysis) can help identify specific areas that need improvement, while still maintaining the privacy of school districts. It also allows us to continue to examine the rural versus urban/suburban areas [47]. We accessed multiple years of data among 6th, 8th, 10th and 12th graders across all available schools. However, the participants were anonymous and we were not able to match or track them over time. We were only able to observe changes in schools generally. The 10th grade seems to be where the jump in suicidal risk is highest. However, it is important to acknowledge that it could be 9th grade, which is not gathered during that year in school. Another limitation due to the anonymous data is the inability to track individuals through the years; is it possible that one individual may have taken the survey in more than one of the survey years and we cannot ascertain the exact impact this has on our results. However, we do note that two years in grade school is a large period of time and the feelings towards many of these questions are shaped as new experiences, evolving values, sexual identity, and enhanced self-consciousness shift from year to year.

Additional limitations are due to the use of standard machine learning techniques: complex tree-based models are harder to examine and cannot establish causal effects from observation data (only controlled studies). Although using SHAP scores allows us to gain insight into the most important features, it does not give a full picture of the complex interactions between variables in the trees (which would be infeasible to describe due to the large number of variables). Another limitation of the SHAP score is that it is relative to the data on which it is trained and thus, no standard cut-off exists to determine how many features to use. However, the first 10 show much greater importance on the predictive curve compared to all others that follow. Thus, although our results have strong predictive value, we cannot assume causation from these results. Furthermore, these results are based only on the Risk and Protective Factor Framework [11]. Future research could involve pairing these datasets with suicide rates in order to assess the relationship between STB and actual suicide rates. Data gathered from across the U.S. would also allow an analysis of location and state influences on adolescent’s STB.

## Conclusion

In this study, a large dataset of adolescent risk and protective factors and SDH data was evaluated using interpretable machine learning methods to predict adolescent STB. Significant predictive power was obtained with the top ten most predictive variables identified—thus establishing a ten variable adolescent risk profile for STB. The study demonstrated that modern machine learning approaches can provide new methods for understanding the correlations between root causes and outcomes, such as STB. The model developed in this study showed significant improvement in predictive accuracy among all PNA questions compared to previous research that used limited adolescent health behavior data. The model’s high accuracy (91%) of predicting those that have STB came from relying on questions that asked whether adolescents were being threatened or harassed through digital media or bullied at school, exposed or involved in serious arguments and yelling at home, their gender, age of first alcohol consumption, feelings regarding school safety, age, and their attitude toward marijuana use. These focused adolescent risk and protective factors of STB identify and affirm targeted areas where policy and prevention programming might be prioritized for the greatest impact.
